# p38α MAPK disables KMT1A-mediated repression of myogenic differentiation program

**DOI:** 10.1186/s13395-016-0100-z

**Published:** 2016-08-22

**Authors:** Biswanath Chatterjee, David W. Wolff, Mathivanan Jothi, Munmun Mal, Asoke K. Mal

**Affiliations:** 1 Department of Cell Stress Biology, CGP-L3-319, Roswell Park Cancer Institute, Elm and Carlton Streets, Buffalo, New York 14263 USA; 2 Present Address: Institute of Molecular Biology, Academia Sinica, Nankang, Taipei, 11529 Taiwan; 3 Present Address: Department of Biotechnology, Bharathiar University, Coimbatore, 641046 Tamilnadu India

**Keywords:** KMT1A, MyoD, p38α, Skeletal muscle differentiation

## Abstract

**Background:**

Master transcription factor MyoD can initiate the entire myogenic gene expression program which differentiates proliferating myoblasts into multinucleated myotubes. We previously demonstrated that histone methyltransferase KMT1A associates with and inhibits MyoD in proliferating myoblasts, and must be removed to allow differentiation to proceed. It is known that pro-myogenic signaling pathways such as PI3K/AKT and p38α MAPK play critical roles in enforcing associations between MyoD and transcriptional activators, while removing repressors. However, the mechanism which displaces KMT1A from MyoD, and the signals responsible, remain unknown.

**Methods:**

To investigate the role of p38α on MyoD-mediated differentiation, we utilized C2C12 myoblast cells as an in vitro model. p38α activity was either augmented via overexpression of a constitutively active upstream kinase or blocked via lentiviral delivery of a specific p38α shRNA or treatment with p38α/β inhibitor SB203580. Overexpression of KMT1A in these cells via lentiviral delivery was also used as a system wherein terminal differentiation is impeded by high levels of KMT1A.

**Results:**

The association of KMT1A and MyoD persisted, and differentiation was blocked in C2C12 myoblasts specifically after pharmacologic or genetic blockade of p38α. Conversely, forced activation of p38α was sufficient to activate MyoD and overcome the differentiation blockade in KMT1A-overexpressing C2C12 cells. Consistent with this finding, KMT1A phosphorylation during C2C12 differentiation correlated strongly with the activation of p38α. This phosphorylation was prevented by the inhibition of p38α. Biochemical studies further revealed that KMT1A can be a direct substrate for p38α. Importantly, chromatin immunoprecipitation (ChIP) studies show that the removal of KMT1A-mediated transcription repressive histone tri-methylation (H3K9me3) from the promoter of the *Myogenin* gene, a critical regulator of muscle differentiation, is dependent on p38α activity in C2C12 cells. Elevated p38α activity was also sufficient to remove this repressive H3K9me3 mark. Moreover, ChIP studies from C2C12 cells show that p38α activity is necessary and sufficient to establish active H3K9 acetylation on the *Myogenin* promoter.

**Conclusions:**

Activation of p38α displaces KMT1A from MyoD to initiate myogenic gene expression upon induction of myoblasts differentiation.

**Electronic supplementary material:**

The online version of this article (doi:10.1186/s13395-016-0100-z) contains supplementary material, which is available to authorized users.

## Background

Differentiation of myogenic precursor myoblast cells into a multinucleated contractile myofiber is a tightly regulated multistep process essential during development, in adulthood, and is often perturbed in pathological conditions. This process demands a precise, highly coordinated, temporally ordered myogenic gene expression program [1] and proceeds through a feed-forward mechanism [2]. The discovery of MyoD [3], the founding member of the myogenic regulatory factors (MRFs) that include Myf5, MRF4, and myogenin, demonstrated its action as a key master regulator of myoblast determination and subsequent differentiation into myotube [4–8]. Initiation of the myogenic differentiation by MyoD requires cooperation with a number of ubiquitous transcriptional regulatory factors. These include chromatin modifiers that synergistically activate muscle-specific gene expression in myoblasts undergoing differentiation [4–6, 9–14]. Similarly, MyoD also directly interacts with components of the basal transcriptional machinery [15–17]. Understanding these interactions of MyoD provide insight into mechanisms guiding the activation of myogenic gene expression program in myoblasts upon induction of differentiation.

Although MyoD is able to execute the entire differentiation program, it is expressed in undifferentiated myoblasts [5, 7, 18]. Paradoxically, MyoD binding analysis revealed its broad occupancy throughout the genome in myoblasts, indicating that its DNA binding alone is not sufficient to activate myogenic gene expression and drive differentiation [5, 19]. Dissection of myogenic differentiation revealed complex epigenetic control of the gene expression program. Accordingly, switching myoblasts from proliferation into differentiation is underpinned by specific epigenetic events to transition myogenic loci from a transcriptionally repressive to activating state. This is caused by chromatin-associated activities, ranging from covalent modification of histones and myogenic regulatory factors to chromatin remodeling [4, 10, 14, 20, 21]. In growing myoblasts, MyoD-bound muscle-specific genes are generally epigenetically marked for transcriptional repression through trimethylation of histone H3 at Lys^9^ (H3K9me3) and/or Lys^27^ (H3K27me3) on promoter/enhancer regions to control their temporal expression [21–25]. Specifically, we demonstrated that H3K9me3-specifc enzyme KMT1A is recruited to the *myogenin* (*Myog*) promoter by associating with MyoD, which establishes a H3K9me3-mediated repressive chromatin environment to prevent premature differentiation [26]. Subsequent data revealed that displacement of KMT1A from MyoD at the onset of differentiation couples with eradication of the repressive H3K9me3 mark and activation of MyoG, a critical regulator in patterning myogenic differentiation-specific gene expression through a feed-forward network with MyoD [1, 2, 27].

An essential step in establishing active transcription at myogenic loci is the temporal recruitment of specific chromatin modifiers that promote MyoD-orchestrated myogenic gene expression and differentiation [10, 14, 20]. At the onset of myoblast differentiation, predominant signaling pathways such as p38α MAPK and PI3/AKT play a major role in transitioning from a transcriptionally repressive to permissive chromatin environment for MyoD-initiated myogenic differentiation program [28–32]. By contrast, p38γ MAPK represses myogenic differentiation by facilitating the association of MyoD with KMT1A in growing myoblasts [33]. Recent studies have also indicated that it is critical to remove repressive H3K9me3 and H3K27me3 marks from myogenic promoter/enhancer regions upon reception of the differentiation-promoting signals. This is carried out by histone demethylases, including JMJD2A [34] and UTX [35]. However, the signaling pathway(s) that control the removal of KMT1A from MyoD, thereby antagonizing KMT1A-dependent H3K9me3 and facilitating the switch from repression into activation of myogenic genes at the onset of differentiation, remains enigmatic.

Here, we identify that p38α MAPK disables MyoD-KMT1A association by phosphorylating KMT1A, thereby permitting a switch from transcriptionally repressive H3K9me3 to active H3K9 acetylation (H3K9ac) [36] in the promoter of important muscle differentiating regulator MyoG. Interference of p38α activity or expression inhibited phosphorylation of KMT1A and prevented its release from MyoD, resulting in sustained H3K9me3-mediated repression of *Myog* and impaired myogenic differentiation. Conversely, forced activation of p38α releases KMT1A from MyoD, resulting in MyoG expression and differentiation. Therefore, this study unveils a new role for p38α as an essential signaling effector of KMT1A phosphorylation to unleash its association with MyoD and initiate myogenic gene activation and differentiation.

## Methods

### Cell culture

Human 293A and 293FT and mouse C2C12 myoblasts have been used previously [26, 37, 38]. C2-4RE-luc reporter cells expressing MyoD-responsive 4RE-luc luciferase gene in C2C12 have been described previously [37]. Human primary skeletal myoblast cells (HsMB) were purchased from Lonza. Except C2C12, C2-4RE-luc, and HsMB, all cells were cultured in DMEM medium containing 10 % FBS supplemented with antibiotic-antimycotic (Invitrogen). C2C12 myoblasts were cultured in growth medium (GM, 20% FBS) and induced to differentiate by switching in differentiation media (DM) medium as described previously [26]. HsMB cells were cultured in growth medium (SKGM-2BulletKit, Lonza) and induced to differentiate by switching to DM. For p38α/β MAPK or PI3K/AKT inhibition studies, SB203580 (SB) and LY294002 (LY) (Calbiochem) were added directly to DM at a final concentration of 5 and 20 μM, respectively. For Flag-KMT1A, HA-MKK6EE, or HA-MKK6DN overexpression studies, cells were transduced with lentivirus expressing with indicated gene or without (empty). Likewise, for knockdown of KMT1A or p38α, lentivirus expressing respective shRNA or random scramble shRNA was transduced into the cells. All cells were grown at 37 °C, 5 % CO_2_ in a humidified atmosphere.

### Lentiviral production and transduction

Lentiviruses were produced in 293FT cells as previously described [26]. Briefly, cells were transfected with lentiviral vector along with packaging vectors using Pure-Fection transfection reagent (System Biosciences). Virus-containing supernatants were collected and filtered. Viruses were diluted with growth medium and transduced three consecutive days in the presence of 8 μg/ml of polybrene (Sigma-Aldrich). Where applicable, virus-transduced cells were subjected to selection against puromycin (1–2 μg/ml) for 2–3 days.

### Vectors and antibodies

Lentiviral pLV vector expressing Flag-KMT1A [38] and LV-HA-MKK6EE and pLV-HA-MKK6DN were generated by subcloning inserts from pcDNA-HA-MKK6EE and pcDNA-HA-MKK6DN (provided by Dr. L. Puri) [39] into pLV vector. For expression of shRNA, KMT1A, p38α, or scramble shRNAs are cloned individually into lentiviral pLKO.1-TRC vector (Addgene) and sequence verified. The shRNA sequences for KMT1A and scramble were described previously [38]. The sequence for p38α shRNA was 5′-AGCCCAGCAACCTAGCTGTTT-3′. Vectors pGEX-4T-3-H3(N) [26] and pGEX-ATF2 (provided by Dr. J. Han) [40] express GST fusion N-terminal histone H3 and ATF2 proteins, respectively.

Antibodies used were phospho-p38 (Cell Signaling 9215), β-actin-peroxidase (Sigma A3854), Flag-M2 (Sigma F3165), myogenin (BD Pharmingen 556358), KMT1A (Cell Signaling 8729, and Millipore 07-550 and 05-615), MyoD (Santa Cruz sc-760 and BD Pharmingen 554130), p38α (Cell Signaling 9790), HA-peroxidase (Sigma H6533), acetyl-histone H3 (Millipore, 06-599), trimethyl-histone H3 (Lys-9) (Millipore 07-442), trimethyl-histone H3 (Lys27) (Millipore 07-449), GAPDH (Biodesign H86504M), Brg-1 (Santa Cruz sc-10768), p21^cip1^ (Santa Cruz sc-397), myosin heavy chain (Developmental Studies Hybridoma Bank, MF-20), total p38 (Cell Signaling 9212), and normal rabbit IgG (Santa Cruz sc-2027).

### Cell extracts, immunoprecipitation, and western blot analysis

Preparation of cell extracts, immunoprecipitation, and western blot analysis were described previously [18]. For both immunoprecipitation coupled or direct western blot analysis, the signal was detected using ECL reagents (GE Healthcare) and the image was retrieved and analyzed by Alpha Innotech FluorChem® HD2 Imager (R&D systems).

### Purification of protein and reporter luciferase assay

Purification GST and GST fusion H3(N) and ATF2 fusion proteins from bacteria were carried out as previously described [18]. Reporter luciferase assay was performed as described [37]. Luciferase activity was determined using the luciferase assay system (Promega) and normalized with protein concentration determined by DC protein assay reagent (Bio-Rad).

### Histone methyl transferase (HMT) activity and kinase assays

The HMT activity assay was performed as described previously [26]. Briefly, control normal IgG or anti-MyoD immunoprecipitates retrieved from equivalent amount of cells extracts were subjected to HMT assay buffer supplemented with 2.5 μg bacterially purified GST-H3(N) and 1.0 μCi of [^3^H]SAM and incubated for 1 h at 30 °C. Reaction mixtures were resolved by SDS-polyacrylamide gel electrophoresis followed by fluorography and autoradiography.

The kinase assay was performed as described previously [41]. In brief, control normal IgG or anti-phospho-p38 immunoprecipitates captured from equivalent amount of cell extracts were incubated with kinase assay buffer in the presence of 10 μCi [γ-^32^P]-ATP with bacterially purified GST-ATF2 substrate for 1 h at 30 °C. To monitor KMT1A phosphorylation directly by p38α, kinase assay was performed by incubating anti-Flag-M2 immunoprecipitates, which was retrieved from 293A cell extracts expressing with or without ectopic Flag-KMT1A, supplemented with purified active p38α (Millipore 14-587) and [γ-^32^P]-ATP in the presence or absence of SB. For control, kinase assay was performed as above using GST-ATF2 as substrate supplemented with purified active p38α.

### Chromatin immunoprecipitation (ChIP) assay

ChIP assays were performed in C2C12 cells grown in GM or in DM for 2 days in the presence and absence of SB according to the method as described previously [26]. ChIP also performed after overexpression of MKK6EE or empty in C2C12 cells treated with SB in GM. Briefly, equivalent amount of chromatin samples (normalized by A260) prepared from HCHO-crosslinked cells were subjected to immunoprecipitates with specific antibody or control normal IgG followed by DNA purification and semi-quantitative PCR analysis or the E-box sites located at the promoter regions of *Myog* and *MyHCIIB* using specific primers. As an input control for ChIP, PCR was performed with DNA from chromatin prior to immunoprecipitation (0.03 % total chromatin used for ChIP) using primers that also amplify the same promoter regions of the above genes. Primers used for *Myog* were described previously [26]. For MyHCIIB promoter regions [42], primers used were as forward: 5′-CACCCAAGCCGGGAGAAACAGCC-3′ and reverse:

5′-GAGGAAGGACAGGACAGAGGCACC-3′. PCR products were fractionated by polyacrylamide gel electrophoresis and visualized by autoradiography.

### Immunofluorescence

Immunofluorescence was performed as previously described [18]. In brief, cells grown in 35-mm culture dishes were fixed with chilled methanol (20 %), rehydrated, blocked with 3 % BSA, washed with PBS containing 0.1 % NP-40, and then incubated with anti-MyHC (MF20) antibody. Alexa-Fluor 488 conjugated secondary antibody (Invitrogen) was used to detect the bound primary MF20 antibody. Images were acquired with a ×10 objective lens using fluorescence microscope Leica DMI 4000 B (Leica microsystems).

## Results

### Activation of p38α abolishes MyoD-associated H3 methyltransferase activity during differentiation

**Fig. 1:**
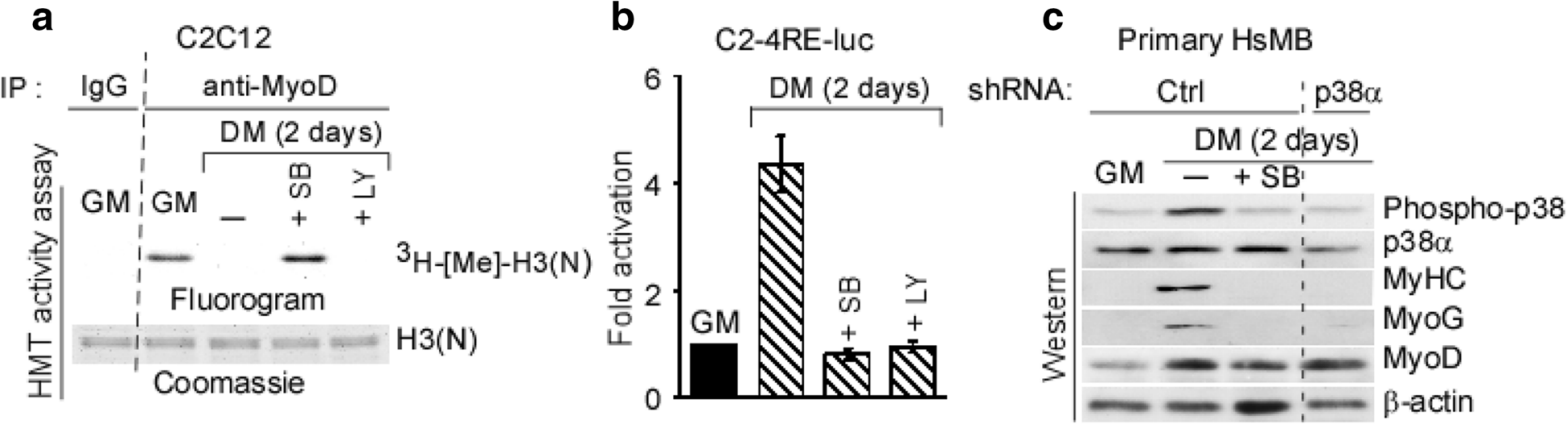
Pharmacological blockade of p38α signaling precludes unleashing of MyoD-associated HMT activity during myoblasts differentiation. **a** MyoD or control IgG immunoprecipitates retrieved from cell extracts of C2C12 grown in GM or DM with or without SB or LY were subjected to HMT activity assay. Methylated and input H3(N) were detected by fluorography and Commassie, respectively. **b** Luciferase activity was assessed in C2-4RE-luc reporter cells grown in GM or DM without or with SB or LY. Luciferase activity was expressed after protein normalization as fold activation. Error bar, ±SEM (*n* = 3). **c** Western blot analysis of primary HsMB cells expressing shRNA of scramble control shRNA (Ctrl) or p38α via lentiviral delivery grown in GM or DM with or without SB, probed with antibodies for indicated proteins. β-actin served as loading control

We demonstrated that KMT1A directly interacts with MyoD, leading to MyoD-associated histone methyltransferase (HMTase) activity which impedes myoblast differentiation, and that release of KMT1A from MyoD abolishes this HMTase activity to promote differentiation [26]. Since differentiation-activated p38α and PI3/AKT signaling cascades promote MyoD-mediated myogenic gene expression [29, 31, 43–45], we asked whether either of these signaling pathways are involved in this removal of KMT1A from MyoD during myoblast differentiation. Hence, we examined MyoD-associated HMTase activity to monitor KMT1A interaction with MyoD in C2C12 myoblasts cultured in GM or DM with either vehicle, p38α/β inhibitor SB20325 (SB), or PI3/AKT inhibitor LY294002 (LY). Results show a loss of MyoD-associated KMT1A activity in differentiated (DM) but not in growing myoblasts (GM) as demonstrated previously by us (Fig. 1a) [26]. Intriguingly, this MyoD-associated activity persists in SB but not in LY-treated C2C12 cells grown in DM. The observed disparity of MyoD-associated KMT1A activity was not due to an unequal amount of substrate revealed by coomassie of histone H3(N) [26] or a variation of MyoD protein levels, which are not altered in C2C12 cells exposed either with SB or LY [31], and verified in immunoprecipitates by western blot analysis (data not shown). Inhibition of p38α/β by SB or PI3K/AKT by LY represses MyoD myogenic activity on target genes at chromatin levels during myoblasts differentiation [31, 46]. Consistently, results show the repression of chromatin-integrated MyoD-responsive 4RE reporter luciferase gene activation in C2C12-derived C2-4RE-luc reporter cells [47] grown in DM treated either with SB or LY (Fig. 1b), assuring the inhibition of the respective pathways by SB or LY in our experimental setting. Together, these findings strongly suggest that activation of p38α/β, but not the PI3/AKT pathway, enables the removal of MyoD-associated KMT1A activity in C2C12 myoblasts induced to differentiate. Although SB treatment inhibits p38α/β isoforms to prevent myogenic gene expression and differentiation of C2C12 cells [44, 48, 49], functional analysis has demonstrated that these effects are mediated exclusively by p38α in these cells and in mice [29, 50]. We evaluated p38α-specific contributions in HsMb differentiation following its downregulation via lentiviral delivery of p38α shRNA compared to control shRNA (Ctrl) (Additional file 1: Figure S1). For this purpose, we examined the protein expression levels of differentiation-associated muscle-specific genes, such as early MyoG and late myosin heavy chain (MyHC), in HsMb cells expressing p38α shRNA induced to differentiate in DM. Cells expressing Ctrl shRNA were cultured in GM or DM with vehicle or SB. Results show inhibited expression of these muscle-related genes in both SB-treated and p38α-depleted cells compared to control cells in DM (Fig. 1c). Results also confirmed the inhibition of p38 activation in SB-treated cells cultured in DM, as phospho-p38 but not total p38α was suppressed in these cells, similar to control cells cultured in growth conditions (GM). As expected, p38α depletion is correlated with decreased phospho-p38 levels in these cells grown in DM. Moreover, MyoD protein levels were detected in all cells cultured in DM. Thus, the anti-myogenic effect of SB, and the persistent MyoD-associated KMT1A activity in SB-treated cells (Fig. 1a), is mainly due to blockade of p38α activation, suggesting that its activation displaces KMT1A activity from MyoD during muscle differentiation.

### Forced p38α activation suppresses KMT1A overexpression-inhibited MyoD activity

**Fig. 2:**
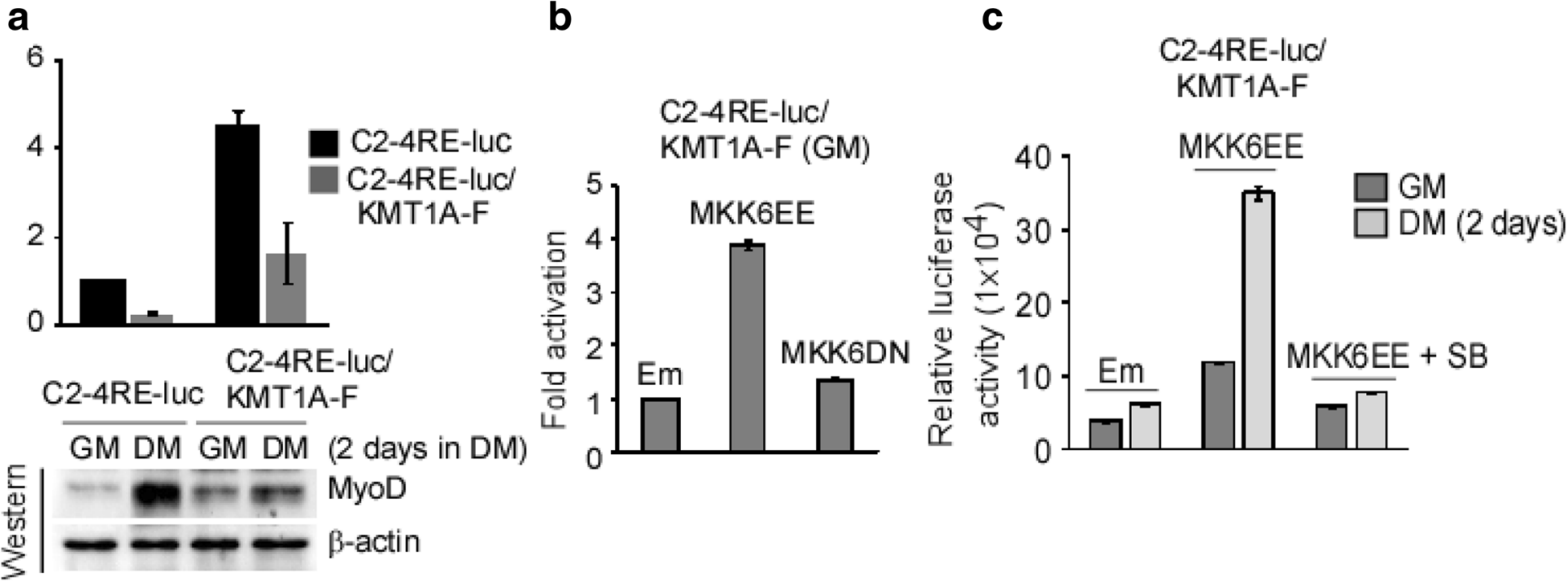
MKK6EE-p38α activation can abolish KMT1A inhibited gene activation by MyoD. **a** C2-4RE-luc/KMT1A-F cells were generated by overexpression of Flag-KMT1A in C2-4Re-luc reporter cells by lentiviral delivery. Subsequently, luciferase activity was assessed in C2-4RE-luc and C2-4RE-luc/KMT1A-F cells in GM or DM and expressed protein after normalization as fold activation. Western blot analysis of these reporter cells extracts probed with antibodies to detect MyoD and β-actin for loading control. **b** Luciferase activity was assessed in C2-4RE-luc/KMT1A-F cells following expression of MKK6EE, MKK6DN or vector control (Em) via lentiviral delivery grown in GM and expressed after protein normalization as fold activation. **c** C2-4RE-luc/KMT1A-F cells expressing vector control (Em) or MKK6EE via lentiviral delivery were grown in GM or DM and MKK6EE-transduced cells treated with or without SB. Afterward, luciferase activity was assessed in these cells and values expressed after protein normalization. Where appropriate, error bar, ±SEM (*n* = 3)

Studies have shown that activation of the p38 pathway, by forced expression of a constitutively active allele of its upstream kinase MKK6 (MKK6EE) [40, 51], activates MyoD-regulated gene transcription in myoblasts cultured in conditions non-permissive for differentiation such as GM media [2, 31, 44]. Since KMT1A overexpression can repress this activity of MyoD during C2C12 differentiation [26], we asked if MKK6EE overexpression can overcome the repressive effect of KMT1A on MyoD. Accordingly, Flag-tagged KMT1A overexpressing C2-4RE-luc reporter cells (C2-4RE-luc/KMT1A-F) were generated via lentiviral delivery. Western blot analysis verifies the expression of Flag-KMT1A along with an increase of total KMT1A and endogenous MyoD levels in these cells compared to parent C2-4RE-luc cells (Additional file 2: Figure S2A). KMT1A overexpression-induced inhibition of MyoD activity was verified by its responsive 4RE reporter gene activation and differentiation-induced MyoD expression in C2-4RE-luc/KMT1A-F cells relative to C2-4RE-luc cells in DM (Fig. 2a). We then overexpressed HA-tagged MKK6EE (herein referred as MKK6EE) in this cell background via lentiviral delivery and measured its effect on MyoD-responsive reporter gene activation compared to cells transduced with empty (Em) vector control lentivirus. Data shows that MKK6EE was sufficient to induce MyoD activity in C2-4RE-Luc/KMT1A-F cells in GM (Fig. 2b), similarly to its effect in parent C2-4RE-Luc reporter cells (Additional file 2: Figure S2B). Moreover, the data shows that overexpression of a dominant negative form of MKK6 (MKK6DN, also tagged with HA) via the same manner fails to induce MyoD-responsive reporter gene activation in these cells grown in GM (Fig. 2b and Additional file 2: Figure S2B), indicating that this induction is dependent on the kinase activity of MKK6 and not the protein directly. Importantly, the effect of MKK6EE on MyoD-mediated reporter gene activation was inhibited following SB treatment in C2-4RE-Luc/KMT1A-F cells grown either in GM or DM (Fig. 2c), suggesting that this induction of MyoD activity is indeed dependent on p38α activation in these cells. Taken together, these data demonstrate that upstream activation of MKK6-p38α signaling can overcome KMT1A-mediated repression of MyoD activity during myoblast differentiation.

### Elevated p38α activity can overcome KMT1A overexpression impeded differentiation

**Fig. 3:**
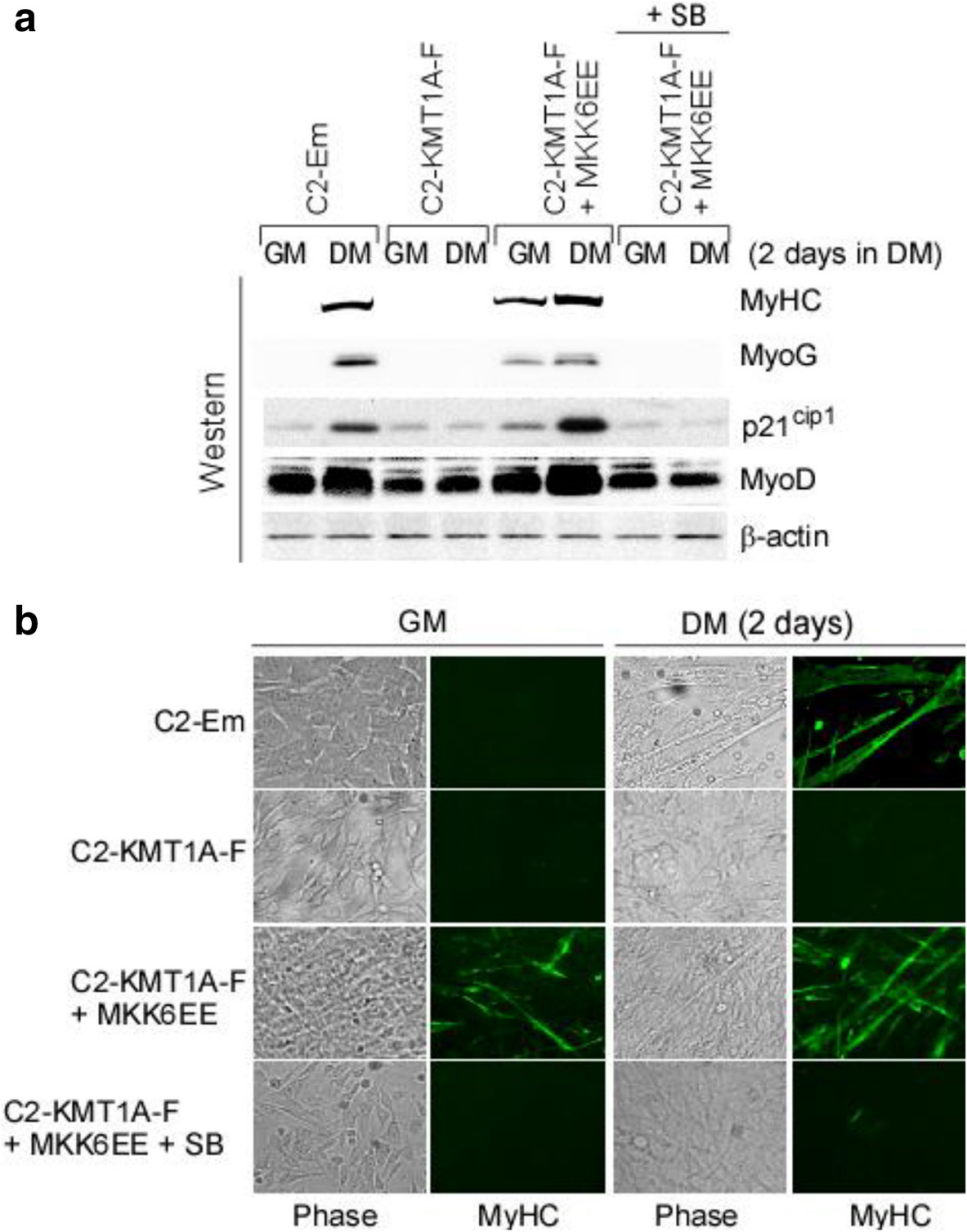
MKK6EE-p38α activation can rescue KMT1A suppressed muscle differentiation. **a** Western blot analysis of cell extracts from vector control C2-Em and Flag-KMT1A overexpressing C2-KMT1A-F cells generated via lentiviral delivery and the same cells after MKK6EE expression by same delivery system grown in GM or DM with or without SB. Probed with antibodies for indicated proteins where β-actin served as loading control. **b** Same as **a** except for western blot analysis, cells were fixed and evaluated for morphology by phase contrast microscopy and immunostained with anti-MyHC antibody (MF20)

Since elevated p38α activity is sufficient to restore KMT1A-inhibited MyoD activity in C2-4RE-Luc/KMT1A-F cells (Fig. 2b, c), we asked if this finding would be corroborated by phenotypic differentiation, which is impaired by KMT1A overexpression in C2C12 cells as previously demonstrated by us [26]. Consistently, results show that Flag-KMT1A overexpression in C2C12 cells (C2-KMT1A-F) via lentiviral delivery inhibited the differentiation related expression of MyHC, MyoG, MyoD, and p21^cip1^ proteins levels relative to vector control virus-transduced cells (C2-Em) grown in DM (Fig. 3a). However, elevation of p38 activation via MKK6EE overexpression resulted in induced levels of the above proteins in C2-KMT1A-F cells grown in GM and with a greater efficiency in DM. Western blot analysis confirmed MKK6EE-induced activation of p38 as revealed by increased phospho-p38 levels in these cells (Additional file 3: Figure S3). Results also show that this induction of differentiation-related proteins by MKK6EE occurred in a p38α activation-dependent manner. Furthermore, MKK6EE-induced activated phospho-p38 and differentiation-related proteins levels were suppressed in C2-KMT1A-F cells either in GM or DM following SB treatment (Fig. 3 and Additional file 3: Figure S3). Together, these findings suggest that MKK6EE-p38α activation overcomes the impairment of differentiation-related gene activation in C2-KMT1A-F cells on induction of differentiation. We then asked whether these results were reflected in phenotypic morphological differentiation by monitoring the ability of C2-Em and C2-KMT1A-F cells to form multinucleated myotubes and express terminal differentiation marker MyHC via immunofluorescence. The data confirms multinucleated myotube formation and MyHC expression are prevented in C2-KMT1A-F cells compared to C2-Em cells grown in DM (Fig. 3b). Conversely, results show that MKK6EE overexpression was sufficient to revert this phenotypic differentiation block in C2-KMT1A-F cells both in GM and DM, while treatment with SB abolished this effect of MKK6EE. These results indicate that the effect of elevated p38α activity on KMT1A overexpression-imposed block of MyoD activity in C1C12 cells is reflected in the phenotypic differentiation of these cells. Taken together, these data suggest that activation of p38α overcomes KMT1A-mediated repression of MyoD to permit terminal differentiation of myoblasts.

### p38α activation-dependent phosphorylation of KMT1A associates with differentiation

**Fig. 4:**
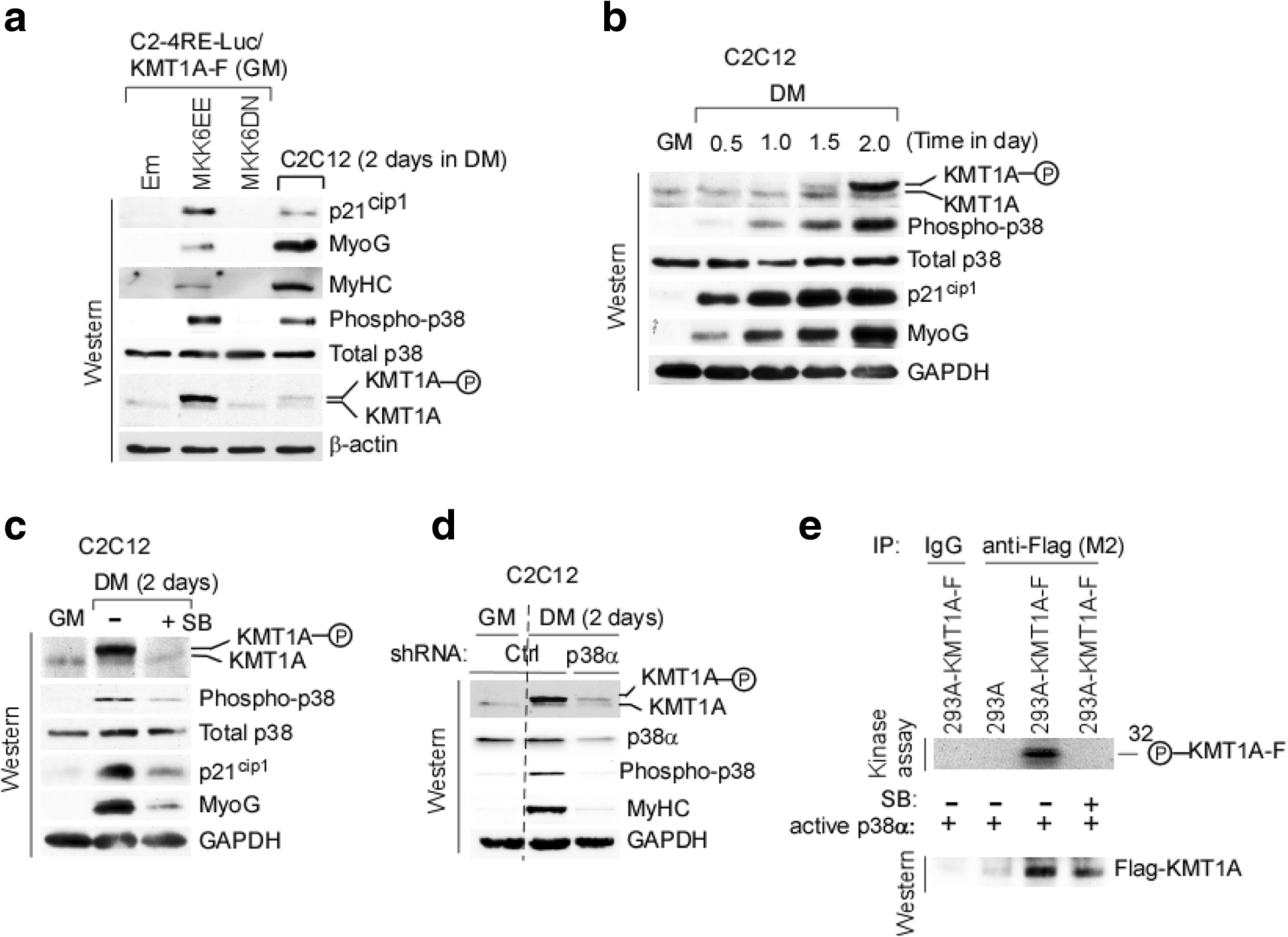
p38α mediates phosphorylation of KMT1A during myoblasts differentiation. **a** Western blot analysis of cell extracts from C2-4RE-luc/KMT1A-F expressing control vector (Em), MKK6EE or MKK6DN via lentiviral delivery grown in GM, and C2C12 grown in DM as positive control of differentiation, probed with antibodies for indicated proteins, and β-actin as for loading control. Phosphorylated KMT1A was detected C2-4RE-luc cells expressing only MKK6EE in GM and C2C12 cells in DM. **b** Western blot analysis of cell extracts from C2C12 grown in GM or DM at various time points, probed with antibodies for detecting indicated proteins, GAPDH as loading control. An increased level of phosphorylated KMT1A was observed with increased activated p38 status during the differentiation of C2C12 myoblasts. **c** Western blot analysis of cell extracts from C2C12 grown in GM or DM in the presence or absence of SB, probed with antibodies against indicated proteins. GAPDH served as loading control. Inhibition of KMT1A phosphorylation was observed by SB-treatment-induced blockade of p38a activation in cells under DM conditions. **d** Western blot analysis of cell extracts from C2C12 cells expressing scramble control shRNA (Ctrl) or p38αshRNA via lentiviral delivery grown as specified in GM or DM, probed with antibodies for indicated proteins, where GAPDH for loading control. Inhibition of KMT1A phosphorylation was observed by p38α knockdown. **e** Control IgG or anti-Flag (M2) immunoprecipitates were retrieved as indicated from extracts of 293A cells expressing with or without Flag-KMT1A via lentiviral delivery. These immunoprecipitates were then subjected to in vitro kinase assay in the presence of purified p38α with or without SB. Subsequently, the reaction mixture was divided equally and evaluated one part for autoradiography and another part for western blot analysis. Phosphorylation of KMT1A directly by p38α was observed

Our previous study demonstrated that KMT1A is phosphorylated during muscle differentiation and hypothesized that differentiation-induced kinase(s) may result in phosphorylation of KMT1A [26]. Since results above clearly indicate that elevated p38α activity overcomes suppression of MyoD activity by KMT1A overexpression to induce differentiation of C2C12 cells (Figs. 2 and 3), we inquired about the status of phosphorylated KMT1A in these cells after altering p38 activity by overexpression of MKK6EE or MKK6DN compared to vector control (Em) via lentiviral delivery. Western blot analysis confirmed that MKK6EE, but not MKK6DN, induced activation of both p38 and muscle differentiation as revealed by induced levels of phospho-p38 and differentiated related p21^cip1^, MyoG, and MyHC compared to control cells cultured in GM (Fig. 4a). C2C12 cells cultured in DM were used as a positive control. Importantly, only MKK6EE overexpression resulted in increased levels of phosphorylated KMT1A, which was also detectable in C2C12 cells cultured in DM, indicating that phosphorylation of KMT1A occurs upon activation of p38 in these cells. This pattern of phosphorylated KMT1A was also observed following re-probing with a separate KMT1A antibody (Additional file 4: Figure S4A). Moreover, this intense band from differentiated C2C12 cells was diminished after depletion of KMT1A via lentiviral shRNA-mediated knockdown (Additional file 4: Figure S4B), confirming that this band is indeed KMT1A. We next sought to determine the kinetics of KMT1A phosphorylation and p38 activation in C2C12 cells induced to differentiate. Western blot analysis shows the appearance of phosphorylated KMT1A at 1.5 days which was more pronounced at 2 days in DM (Fig. 4b). Moreover, phosphorylation of KMT1A strongly correlated with increased levels of activated phospho-p38 and higher expression of p21^cip1^ and MyoG. To test whether this KMT1A phosphorylation is indeed dependent on differentiation-induced p38α activity, C2C12 cells were induced to differentiate in DM after treating cells with or without SB. Western blot analysis shows that SB treatment-induced inhibition of p38 (phospho-p38) blocks the phosphorylation of KMT1A in addition to inhibiting p21^cip1^ and MyoG (Fig. 4c), suggesting that apparently p38α activation directs KMT1A phosphorylation during C2C12 differentiation. To confirm that this effect of SB on KMT1A phosphorylation is through p38α, phosphorylated levels of KMT1A was evaluated by western blot analysis of C2C12 cells expressing control shRNA (Ctrl) grown in GM or induced to differentiate while expressing Ctrl or p38α shRNA in DM. Results show that p38α depletion, resulting in a lack of activated phospho-p38, indeed blocks KMT1A phosphorylation in these cells (Fig. 4d). Moreover, inhibition of MyHC expression in p38α-depleted cells in DM is consistent with previous studies specifically implicating p38α in myogenic differentiation [29, 50]. We also asked whether p38α may be directly responsible for this phosphorylation of KMT1A. Therefore, Flag-KMT1A was overexpressed in 293A cells (Additional file 4: Figure S4C) and immunoprecipitated with anti-Flag (M2) antibodies or control IgG from extracts of cells expressing Em or Flag-KMT1A. Subsequently, an in vitro kinase assay coupled with western blot analysis was performed after the addition of purified active p38α to these immunoprecipitates with or without SB. Results show phosphorylation of immunoprecipitated Flag-KMT1A protein directly by p38α, which is blocked in the presence of SB (Fig. 4e). The activities of both p38α and SB were verified by performing a similar kinase assay using bacterially purified known p38 kinase substrate ATF2 (Additional file 4: Figure S4D). Together, these results indicate that phosphorylation of KMT1A associates with differentiation of myoblasts and that this phosphorylation is specifically dependent on the activation of p38α.

### Phosphorylation of KMT1A by p38α relieves MyoD during differentiation

**Fig. 5:**
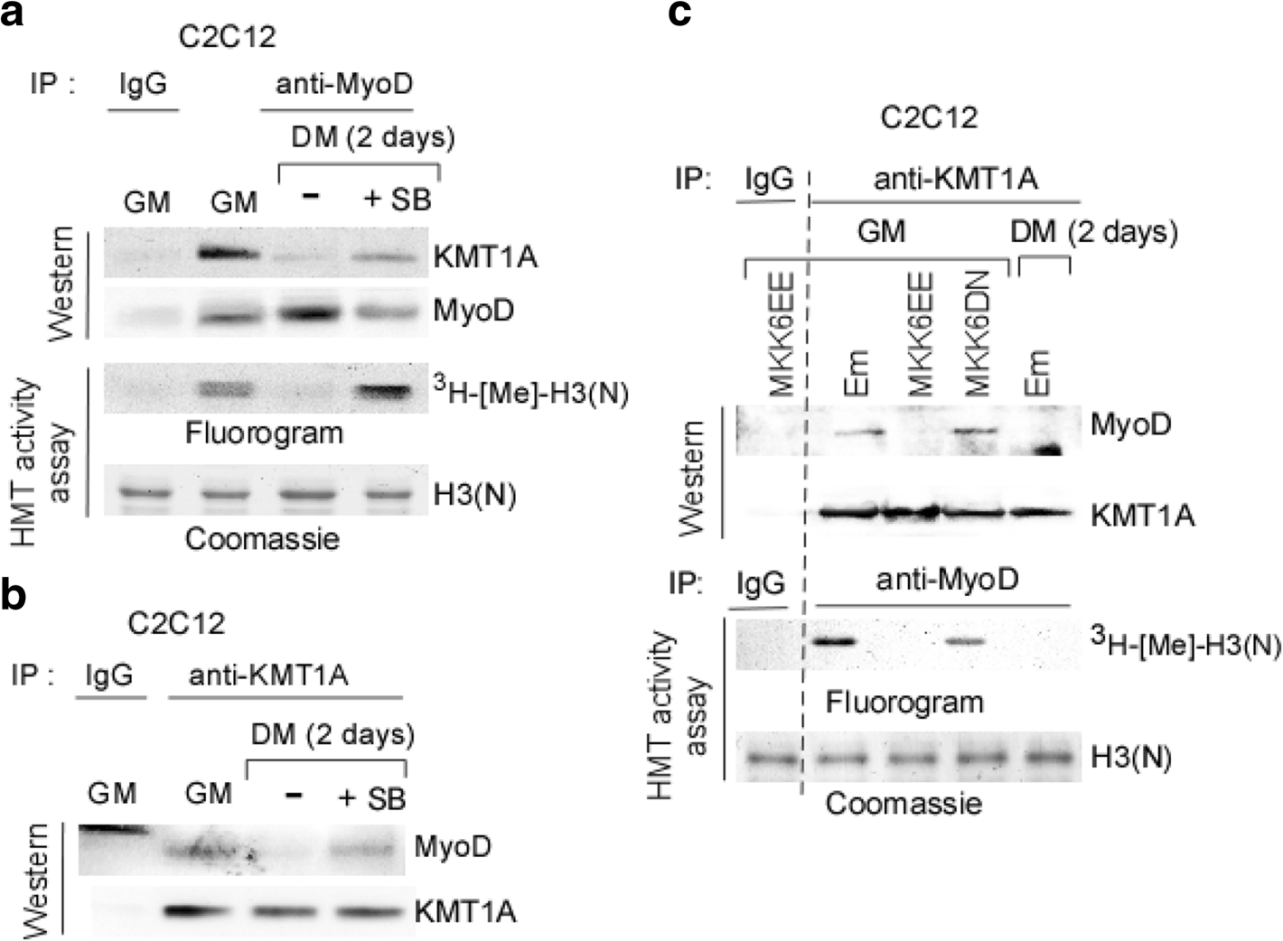
p38α unleashes KMT1A via phosphorylation from MyoD during differentiation. **a** Control IgG or anti-MyoD immunoprecipitates retrieved from cell extracts of C2C12 cells grown GM or DM with or without SB were divided equally and evaluated one part for western blot analysis, probed with antibodies against KMT1A and MyoD. Another part was subjected to HMT activity assay. Methylated and input H3(N) were detected by fluorography and Commassie, respectively. **b** Control IgG or anti-KMT1A immunoprecipitates retrieved from cell extracts used in **a** were subjected to western blot analysis, probed with antibodies against MyoD and KMT1A. **c** Control IgG, anti-KMT1A, or anti-MyoD immunoprecipitates were retrieved from cell extracts of C2C12 cells expressing vector control (Em), MKK6EE or MKK6DN via lentiviral delivery grown in GM or DM. Subsequently, IgG and KMT1A immunoprecipitates were subjected to western blot analysis, probed with antibodies against MyoD and KMT1A. Separately, IgG and MyoD immunoprecipitates were subjected to HMT activity assay. Methylated and input H3(N) were detected by fluorography and Commassie, respectively

Based on the above results, we hypothesized that p38α-mediated phosphorylation of KMT1A results in its release from MyoD, thereby rescuing KMT1A impeded MyoD activity in myoblasts proceeding to differentiation. To test this, we examined MyoD-associated KMT1A and corresponding HMTase activity by analyzing of anti-MyoD immunoprecipitates from C2C12 cells grown in GM or DM without or with SB to block p38α activation. Consistent to our previous study [26], results of western as well as HMTase activity assays show that KMT1A and its HMTase activity are associated with MyoD in cells before (GM) but not after induced to differentiate in DM without SB (Fig. 5a). In contrast, persistent levels of both MyoD-associated KMT1A and HMTase activity were observed in cells that have been in DM with SB. Western analysis also shows no disparity in the levels of MyoD recovered from extracts these cells. Accordingly, western analysis of anti-KMT1A immunoprecipitates shows MyoD association with KMT1A in cells grown in GM or DM with, but not without, SB. Western also confirms that an equivalent level of KMT1A recovered from these cells (Fig. 5b). Together, these findings show that activated p38α activity is necessary to remove KMT1A from MyoD at the induction of C2C12 cell differentiation.

We then asked whether elevated p38α activity was sufficient to remove KMT1A from MyoD at the onset of myoblast differentiation. To address this, we examined KMT1A association with MyoD after elevating p38α activity by MKK6EE overexpression via lentiviral delivery in C2C12 cells grown in GM by western blot analysis. As controls, cells overexpressing MKK6DN or empty vector control (Em) via the same manner were grown in both GM and DM, respectively. The analysis shows MyoD in anti-KMT1A immunoprecipitates from above Em control cells in GM but not in DM, as anticipated (Fig. 5c). Remarkably, this recovery of MyoD was not observed in cells overexpressing MKK6EE in GM although comparable levels of KMT1A were recovered from extracts all these cells, indicating that MKK6EE-p38α activation displaces KMT1A from MyoD from cells even in conditions non-permissible for differentiation. To test if this presence or absence of MyoD with KMT1A is mirrored to its activity associated with MyoD, an in vitro HMTase activity assay was performed in anti-MyoD immunoprecipitates retrieved from these cells extracts. Results show the loss of MyoD-associated activity in GM only in MKK6EE overexpressing cells. Western blot analysis (Additional file 5: Figure S5A) confirmed the protein expression of both MKK6EE and MKK6DN, which are tagged with HA as described above (Fig. 2), in these cells. Results of an in vitro kinase assay performed in anti-phospho p38 immunoprecipitates using bacterially purified GST-ATF2 protein as for substrate also confirm elevated p38α activity in cells overexpressing MKK6EE but not MKK6DN or vector control cells cultured in GM and activated p38α in vector control cells in DM as expected (Additional file 5: Figure S5B). Taken together, these results demonstrate that elevated p38α activity results in KMT1A phosphorylation and its dissociation from MyoD during myoblast differentiation.

### Activation of p38α eradicates KMT1A-directed H3K9me3 marks at the *myogenin* promoter

**Fig. 6:**
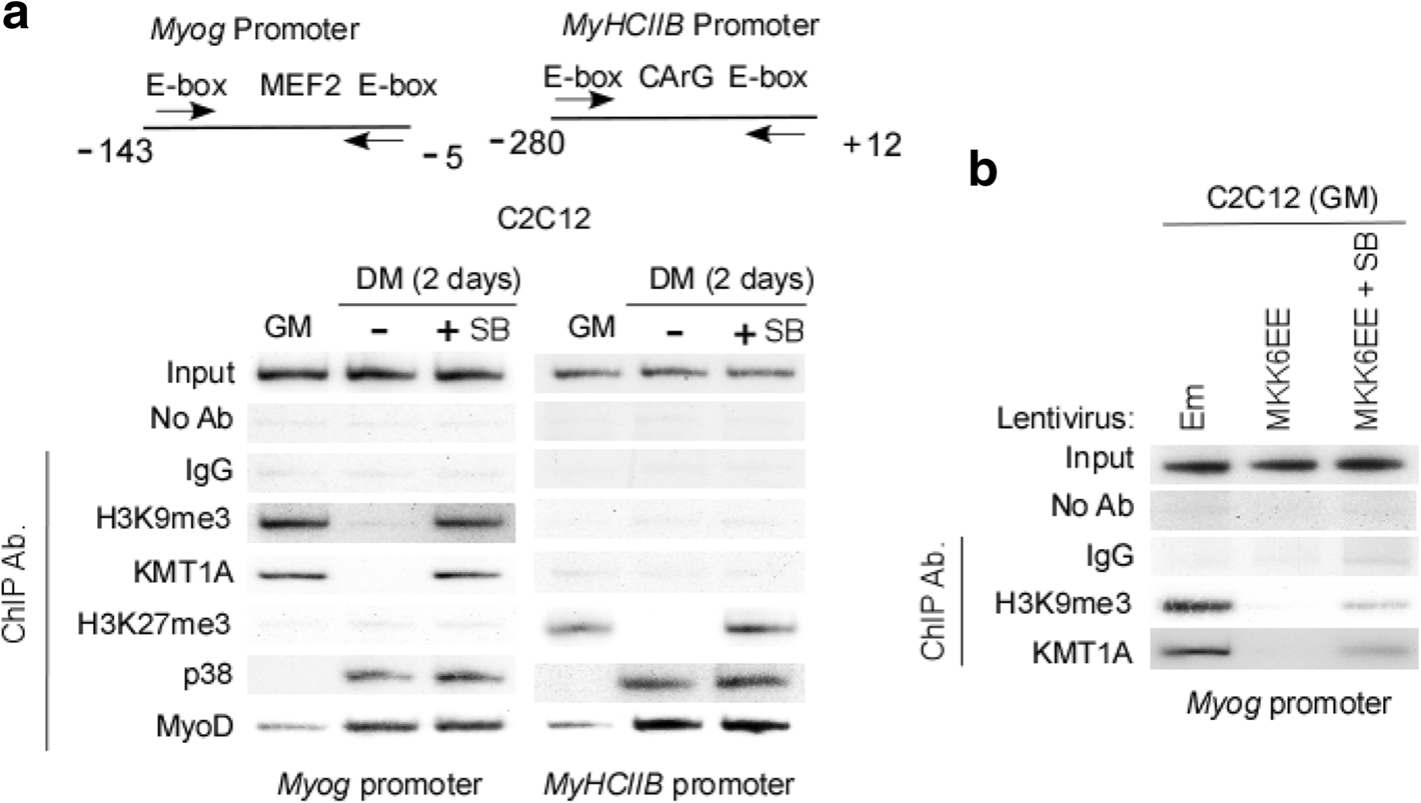
Inhibition of p38α activation retains KMT1A on chromatin of the *myogenin* promoter during differentiation. **a** ChIP analysis of E box-containing regions within the *Myog* and *MyHCIIB* promoters was performed on chromatin of C2C12 cells gown in GM or DM treated with or without SB. Schematic diagram represents E box-containing regions of these genes promoters and arrows indicate primers used for PCR reaction. ChIP was performed with antibodies against proteins as indicated, where no antibodies or IgG were used as controls. **b** ChIP analysis of same regions of Myog promoter was performed on chromatin of C2C12 cells expressing vector control (Em) or MKK6EE vial lentiviral delivery grown in GM in the presence or absence of SB. ChIP was performed with anti-H3K9me3 or anti-KMT1A antibodies, where no antibodies or IgG were used as controls

Results above demonstrate that p38α-dependent phosphorylation of KMT1A results in its removal from MyoD and myogenic differentiation. We asked whether this event contributes to alterations in chromatin at the promoters of myogenic genes relevant to differentiation. We primarily focused on the promoter of *Myog*, at which MyoD-associated KMT1A establishes transcriptionally repressive H3K9me3 marks prior to activation of differentiation [25, 26]. We sought to determine if p38α activation-associated MyoG expression, and in turn differentiation, is controlled through eradication of KMT1A-directed H3K9me3 from the promoter of this gene. Therefore, ChIP assay was performed for H3K9me3 and KMT1A levels on *Myog* promoter in C2C12 cells grown in GM or DM treated without or with SB. Results show that a decrease in H3K9me3 is accompanied by a loss of KMT1A levels in C2C12 cells upon induction to differentiate by switching from GM into DM (Fig. 6a, left panel), as reported previously [26]. Importantly, the levels of H3K9me3 and KMT1A at the *Myog* promoter are persistent in SB-treated cells grown in DM, indicating that the loss of H3K9me3 and KMT1A at this promoter is dependent on p38α activation during the differentiation. Consistent with previous studies [22, 23], we failed to observe the repressive H3K27me3 marks at the *Myog* promoter in growing C2C12 myoblasts (GM). Results of ChIP analysis further show that increased binding of MyoD and p38α at this gene promoter was not substantially altered by blockage of p38α activation in C2C12 cells treated with SB in DM, in agreement with previous studies [30, 31]. In addition to *Myog* promoter, ChIP assay was performed for these conditions at the promoter of *MyHCIIb*, a lately expressed myogenic gene targeted for H3K27me3-mediated repression [23] which utilizes the combined activity of MyoD and MyoG for its acivation [27]. In contrast to MyoG, results show no enrichment of KMT1A or H3K9me3. However, consistent with described study, H3K27me3 was detected on *MyHCIIb* promoter in myoblasts and retained in SB-treated cells induced to differentiate in DM (Fig. 6a, right panel). Further, we found that both MyoD and p38α were enriched on *MyHCIIb* promoter on induction of differentiation and remained unchanged following SB treatment. To confirm the specificity of ChIP assay, PCR was performed on non-muscle *amylase* promoter and detected no binding for the above investigations (data not shown). These results indicate that the p38α activity eradicates KMT1A, thereby depleting its restrictive H3K9me3 mark from the *Myog* promoter during myoblast differentiation. We further tested whether elevated p38α activity by MKK6EE is sufficient to remove the KMT1A-mediated H3K9me3 marks from the *Myog* promoter in growing myoblasts. Therefore, ChIP assay was performed for determining the levels of KMT1A and H3K9me3 on the promoter of *Myog* after MKK6EE overexpression via lentiviral delivery in C2C12 cells cultured in GM. Strikingly, both KMT1A and H3K9me3 are lost from the *Myog* promoter in C2C12 cells expressing MKK6EE but not vector control (Em), while treatment with SB partially recovered their loss in MKK6EE-expressing cells (Fig. 6b). Collectively, these results demonstrate that p38α activation dampens repressive H3K9me3 marks from *Myog* in part by removing KMT1A bound with MyoD at the onset of C2C12 cells differentiation.

### Activation of p38α permits histone H3 lysine 9 acetylation at the *myogenin* promoter

**Fig. 7:**
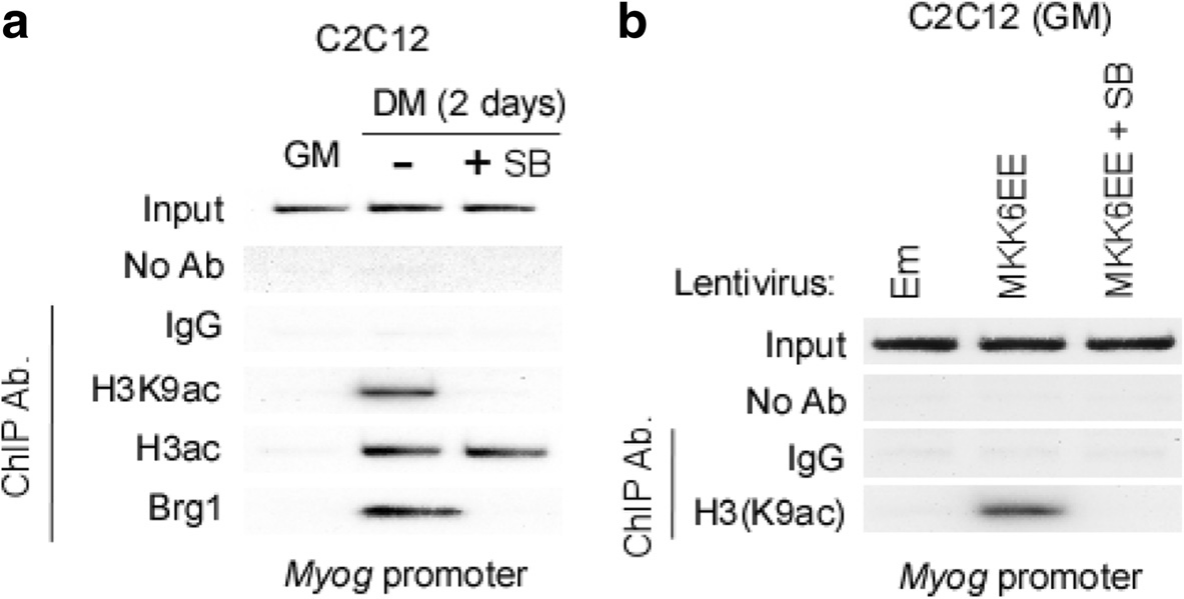
Blockade of p38α precludes acetylation at histone H3 lysine 9 on the chromatin of *myogenin* promoter during differentiation. **a** ChIP analysis of E box-containing promoter region of *Myog* was performed on chromatin used in Fig. 6a with indicated anti-acetylated histone H3 and Brg1 antibodies. In parallel, Input and IgG or no antibodies as controls were processed. **b** Similar to **a**, except ChIP performed with anti-acetylated H3 lysine 9 antibodies on chromatin used in Fig. 6b

Activation of *Myog* is accompanied with a switching from repressive methylation to activating acetylation of histone H3 at the promoter of this gene during myoblast differentiation [24, 25, 31]. Since methyl/acetyl modifications fail to occur concurrently at the same lysine residue, we tested whether preclusion of KMT1A-mediated H3K9 methylation by p38α activity (Fig. 6) permits acetylation of H3 at this residue (H3K9ac) on the promoter of *Myog* to promote its activation during myoblasts differentiation. Therefore, ChIP assay was performed for H3K9ac on the *Myog* promoter in C2C12 cells grown in GM or DM with or without SB treatment. Consistent to our previous study [24], results show an enrichment of H3K9ac on the promoter *of Myog* in differentiating C2C12 cells grown in DM (Fig. 7a). However, this enrichment was not observed in cells exposed with SB. Studies have demonstrated that the chromatin recruitment of histone acetyltransferases p300/PCAF increases global H3 acetylation (H3ac) at the *Myog* promoter on induction of C2C12 cells differentiation [24, 31] and that these activities were not affected after p38α inhibition in cells treated with SB [31]. Our results also show no alteration of total H3ac on the *Myog* promoter in C2C12 cells following SB treatment in DM, suggesting that the blockade of p38α precluded specifically H3K9ac at this promoter (Fig. 7a). To confirm that p38α activation was blocked, we performed ChIP assay for Brg1, a critical component of SWI/SNF chromatin remodeling complex recruited to the *Myog* promoter in a p38α activity-dependent manner [31]. Results show as reported previously that Brg1 was recruited to the *Myog* promoter of C2C12 cells in DM, while treatment with SB blocked this recruitment (Fig. 7a). Together, these results demonstrate that p38α activity is necessary for the acetylation at H3K9 on the *Myog* promoter at the onset of myoblasts differentiation. We then tested whether p38α activity is sufficient for the above event. Hence, ChIP assay was performed for H3K9ac on the *Myog* promoter in C2C12 cells cultured in GM after expression of MKK6EE via lentiviral delivery followed by treatment without or with SB. Results show the enrichment of H3K9ac at this promoter in cells expressing MKK6EE compared to vector control (Em) and that this increase of H3K9ac is inhibited by SB treatment (Fig. 7b), providing further evidence for p38α activity in orchestrating acetylation of H3K9 on the *Myog* promoter. Collectively, these results establish a critical role for p38α activity in authorizing transcriptionally active H3K9 acetylation at the *Myog* promoter on the induction of C2C12 myoblast differentiation.

## Discussion

In this study, we set out to understand how MyoD can dissociate from KMT1A in proliferating myoblasts to activate MyoD-target genes upon induction to differentiate into myotubes. Here, we conclusively demonstrate an integral role for p38α activity in releasing KMT1A from MyoD to activate muscle gene expression on the onset of differentiation. Specifically, we show p38α activity-dependent phosphorylation of KMT1A during differentiation and that phosphorylated KMT1A releases from chromatin-bound MyoD within the promoter of transcriptionally repressed *Myog* in proliferating myoblasts. This dismissal of KMT1A results in a decline of transcriptionally repressive H3K9me3 mark at the *Myog* promoter and an enrichment of permissive acetylated H3K9 and the expression of MyoG, which plays a critical and unique role in MyoD regulated differentiation program. Thus, our findings establish that p38α-directed uncoupling of MyoD/KMT1A complex ensures the eradication of the repressive H3K9me3 mark, facilitating the formation of a transcriptionally active chromatin state at myogenic loci on the onset of muscle differentiation.

MyoD serves as a central controller of the myogenic gene expression network for skeletal muscle differentiation to proceed [4, 7]. MyoD binds and regulates myogenic genes through repression of these targets in proliferating myoblasts but can be activated only upon receiving signals that launch differentiation [4, 5, 19, 52]. In fact, signals regulating MyoD interactions with cofactors, including chromatin modifiers, are critical for fine-tuning chromatin status of myogenic genes and determining the net outcome [30–33]. We previously demonstrated that assembly of KMT1A through interaction with MyoD at the *Myog* promoter represses its premature expression in proliferating myoblasts [26]. In this context, a study has shown that p38γ supports the interaction of KMT1A with MyoD via phosphorylation [33]. However, it is well known that p38α acts oppositely to p38γ to promote MyoD-mediated myogenic gene expression and differentiation [53, 54], a situation where KMT1A unleashes from MyoD [26]. Interestingly, here we found that KMT1A remains associated with MyoD after inhibition of p38α in myoblasts induced to differentiate, indicating that p38α may directly target KMT1A for disengaging with MyoD, thereby influencing myogenic gene expression. Although p38α activates myogenic gene expression by directly targeting positive chromatin modifier/transcriptional regulator of MyoD [2, 28, 30, 46, 53], we previously demonstrated that KMT1A overexpression entirely abolishes ability of myoblasts to differentiate [26]. Interestingly, results show that KMT1A overexpression moderately affected p38 activation in myoblasts, likely due to failure of these cells to amplify the differentiation signal. In this cell background, we hypothesized that this level of p38α activity may be inadequate to overcome inhibition of myogenic differentiation by KMT1A overexpression, since p38 activity status is critical in deciding the extent of myogenic differentiation [2, 44]. Indeed, we found that MKK6EE-p38 activation, where MKK6EE overexpression results in the activation of primarily p38α [33], is quite proficient in rescuing KMT1A-inhibited MyoD function to induce myogenic gene expression and phenotypic differentiation under both growth and differentiation conditions. Note that KMT1A overexpression does not prevent activation of p38α by MKK6EE, indicating that KMT1A indeed acts downstream of p38α activity. Further, this effect of MKK6EE-p38α was blocked in cells treated with SB and induced to differentiate. Together, these findings suggest that p38α actively participates in disengaging KMT1A-mediated suppression of MyoD-regulated myogenic differentiation.

KMT1A is a phosphoprotein and that phosphorylation modulates its function [55–57]. We found that KMT1A phosphorylation increases in myoblasts on induction of differentiation, a finding consistent to our previous study [26]. Further, we found a strong correlation between differentiation-induced levels of p38 activation and phosphorylated KMT1A status, and that MKK6EE-p38α activation results in increased phosphorylation of KMT1A in myoblasts. This p38α-coupled KMT1A phosphorylation is also clearly reflected in MyoD-regulated gene expression during differentiation. These findings suggest that p38α activity likely mediates the phosphorylation of KMT1A in promoting MyoD function on inducing myoblast differentiation. Studies have indicated that CDK-dependent phosphorylation of KMT1A controls its function during cell cycle progression [55, 57]. Our data show that inhibition of p38α via SB treatment or shRNA-mediated knockdown blocks KMT1A phosphorylation during myoblast differentiation, strongly emphasizing that p38α activity directed KMT1A phosphorylation in this process. Indeed, in vitro kinase assay confirms that KMT1A is a direct target of p38α. Once activated, p38 phosphorylates serine/threonine residues of the substrates. A previous report has indicated that KMT1A is preferably phosphorylated at serine residues and that seven putative phosphorylation sites exist within it. Of these, four are in the C-terminal, one at the beginning of SET and two around the chromo domains [55]. Currently, we are in the process to identify p38α target site(s) on the KMT1A and their impact to myogenic differentiation.

Previously, we demonstrated that KMT1A association with MyoD restrains its function in contributing to proliferation by preventing myogenic differentiation [26]. p38γ-directed phosphorylation of MyoD has been shown to assist this association with KMT1A, thereby influencing this myogenic decision [33]. By contrast, it is well recognized that p38α directly targets positive epigenetic and transcriptional regulators via phosphorylation to allow their assembly and/or association with MyoD to promote myogenic differentiation. For instance, phosphorylation of chromatin-associated factor BAF60c in a MyoD-BAF60c complex facilitates the recruitment of SWI/SNF chromatin remodeling complex to myogenic loci for gene activation [28, 46]. Likewise, p38α phosphorylates MEF2, a transcription factor cooperating with MyoD for both its recruitment and expression of late myogenic genes in a feed-forward mechanism [2, 13, 58], and enforces its recruitment of Ash2L/MLL2 methyltransferase complexes to activate myogenic loci [4, 30]. Additionally, p38α-mediated phosphorylation of Ezh2 methyltransferase, a negative epigenetic regulator of myogenic differentiation [23], has been shown to enhance its interaction with YY1 in repressing Pax7 expression, critical for switching myoblasts from proliferation into differentiation [59]. Interestingly, we found p38α-directed phosphorylation of KMT1A during the transition from proliferation into differentiation of myoblasts. Because the presence of a KMT1A/MyoD complex is restricted to proliferating myoblasts [26], p38α may engage in phosphorylation-mediated removal of KMT1A from MyoD during differentiation. Indeed, data of immunoprecipitation-coupled western or HMTase activity assays show that KMT1A remains associated with MyoD only after blockade of p38α activation on the induction of differentiation, similar to proliferating myoblasts. Conversely, this association of KMT1A with MyoD was abolished in proliferating myoblasts after MKK6EE-p38α activation. Thus, our findings emphasize that p38α releases KMT1A from MyoD via phosphorylation at the onset of myoblasts differentiation, thereby rescuing MyoD from this negative epigenetic regulator of myogenic gene expression. KMT1A has been shown to participate in forming a multimeric complex including histone methyltransferase G9a [60]. In proliferating myoblasts, G9a interacts with MyoD and inhibits its transcriptional activity via methylation to impede myogenic differentiation [61]. It is possible that p38α-directed dissociation of KMT1A might also assist the removal of G9a from MyoD for influencing myogenic gene expression during proliferation to differentiation switch of myoblasts.

Studies have shown that MyoD binds to chromatin of myogenic genes both in proliferating and differentiated myoblasts [5, 19]. However, the decision to repress or activate myogenic gene expression in differentiation depends on the engagement of specific signaling mediators and chromatin modifiers at the myogenic loci [4, 14, 62]. In this context, assembly of KMT1A/MyoD complex by p38γ establishes H3K9me3-dependent transcriptionally repressive chromatin state of *Myog* during myoblast proliferation [26, 33]. It is worth noting that *Myog* is an important myogenic regulator of differentiation, and that its suppression during proliferation is critical to allow for the appropriate expansion of precursor myoblasts. In contrast to p38γ, however, p38α coordinates the assembly of cofactors responsible for achieving permissive chromatin architecture during the switch from proliferation-to-differentiation in myoblasts [28, 30, 31]. A recent study has indicated that p38α exerts this myogenic function, in part, via binding and acting at chromatin [54]. Nonetheless, we demonstrated that the eradication of KMT1A-mediated repressive H3K9me3 methylation is coupled with permissive H3K9ac marked chromatin of *Myog* during myoblasts transition from proliferation-to-differentiation [26]. Of interest, we observed that the blockade of p38α activity during this transition, however, sustains KMT1A engagement along with H3K9me3, and opposes H3K9ac at the chromatin of *Myog* promoter. Alternatively, MKK6EE-p38α activation shows removal of both KMT1A and H3K9me3 coupled with an enrichment of H3K9ac on the *Myog* promoter in proliferating myoblasts. Thus, these findings clearly demonstrate that p38α activity is critical for disengaging the KMT1A-imposed repressive H3K9me3 to facilitate H3K9ac-permissive chromatin state of *Myog* at the onset of myoblasts differentiation.

## Conclusions

Collectively, results in this study uncover p38α-KMT1A signaling as an integral myogenic transcriptional regulatory system in which phosphorylation uncouples KMT1A from MyoD to allow a switch from repressive to permissive chromatin, allowing the initiation of myogenic gene expression during myoblasts differentiation. Given that higher levels of KMT1A contribute to impaired myogenic differentiation in alveolar subtype of rhabdomyosarcoma [38], and that p38 activity is deficient in a majority of rhabdomyosarcoma tumors regardless their subtype [39], it remains to be determined whether and to what extent dysregulated p38γ/α-KMT1A signaling axis contributes to restraining rhabdomyosarcoma differentiation, a question of therapeutic significance.

## Abbreviations

4RE, four right E-box; AKT, V-Akt murine thymoma viral oncogene homolog; ATF2, activating transcription factor 2; ChIP, chromatin immunoprecipitation; DM, differentiation media; GM, growth media; H3K9ac, acetylated histone H3 lysine 9; H3K9me3, tri-methylated histone H3 lysine 9; HMT, histone methyltransferase; HsMB, human primary skeletal myoblast cells; KMT1A, lysine methyltransferase 1A; MAPK, mitogen-activated protein kinase; MKK6, mitogen-activated protein kinase kinase 6; MRF4, myogenic regulatory factor 4; MRFs, myogenic regulatory factors; MyHC, myosin heavy chain; MyoG, myogenin; Pax7, paid box 7; PCAF, p300/CBP-associated factor; PI3K, phosphatidylinositol-4,5-bisphosphate 3-kinase, catalytic subunit alpha; SWI/SNF, switch/sucrose nonfermentable chromatin remodeling complex

## Additional files

Additional file 1: Figure S1. Knockdown of p38α was monitored by western blot analysis of cell extracts from primary HsMB cells expressing control scramble shRNA (Ctrl) or p38α shRNA, probed with antibodies for p38α, total p38, β-actin as loading control. Decreased levels of both p38α and total p38 were observed by p38α shRNA relative to Ctrl shRNA. (PPTX 47 kb) Additional file 2: Figure S2. (A) Ectopic Flag-KMT1A expression was determined by western blot analysis of C2-4RE-luc and C2-4RE-Luc/KMT1A-F cells grown in GM, probed with antibodies to Flag-KMT1A, total KMT1A, MyoD, and β-actin as loading control. Flag-KMT1A expression was observed only in C2-4RE-luc/KMT1A-F cells. (B) Luciferase activity was monitored in C2-4RE-luc cells expressing vector control (Em), MKK6EE or MMK6DN grown in GM and values expressed after protein normalization as fold activation. Error bar, ±SEM (*n* = 3). MyoD-responsive reporter luciferase gene activation was observed by MKK6EE but not MKKDN in these cells. (PPTX 59 kb) Additional file 3: Figure S3. The levels of phosphor-p38 as an indicator of its activated status was monitored by western blot analysis of indicated cells grown in GM or DM in the presence or absence of SB, probed with anti-phospho-p38 and anti-p38 antibodies, where the later antibodies used for monitoring the equivalent levels of total p38 in cell extracts. (PPTX 57 kb) Additional file 4: Figure S4. Western blot analysis verified phosphorylated KMT1A by re-probing the membrane of the western blot results presented in Fig. 4a with a separate KMT1A antibody. (B) Western blot analysis of C2C12 cells expressing control scramble shRNA (Ctrl) or KMT1A shRNA via lentiviral delivery grown in GM or DM, probed with antibodies against KMT1A, and β-actin as loading control. Decreased levels of both under- and phosphorylated KMT1A were observed by KMT1A shRNA relative to Ctrl. (C) Flag-KMT1A overexpression was monitored by western blot analysis of cell extracts following it expression in 293A cells (293A-KMT1A-F) via lentiviral delivery. (D) In vitro kinase assays was performed for p38α activity using GST or GST-ATF2 as substrate by in vitro kinase assays in the presence and absence of SB. Commassie and autoradiography detected inputs GST/GST-ATF2 proteins and phosphorylated ATF2, respectively. (PPTX 99 kb) Additional file 5: Figure S5. Expression of HA-tagged MKK6EE and MKK6DN in C2C12 cells was verified by western blot analysis, probed with antibodies against HA, and β-actin as loading control. (B) Control IgG or anti-phospho-p38 immunoprecipitates retrieved from extracts of indicated cells grown in GM or DM were subjected to in vitro kinase assays to monitor p38 activation using GST-ATF2 as substrate. Autoradiography and Commassie detected phosphorylated ATF2 and GST-ATF2 protein, respectively. (PPTX 75 kb)
