# Supplementary figures and images for: p38α MAPK disables KMT1A-mediated repression of myogenic differentiation program
Source: Skelet Muscle. 2016 Aug 22;6:28. doi: 10.1186/s13395-016-0100-z (PMC4993004; doi:10.1186/s13395-016-0100-z)

## Slide 1
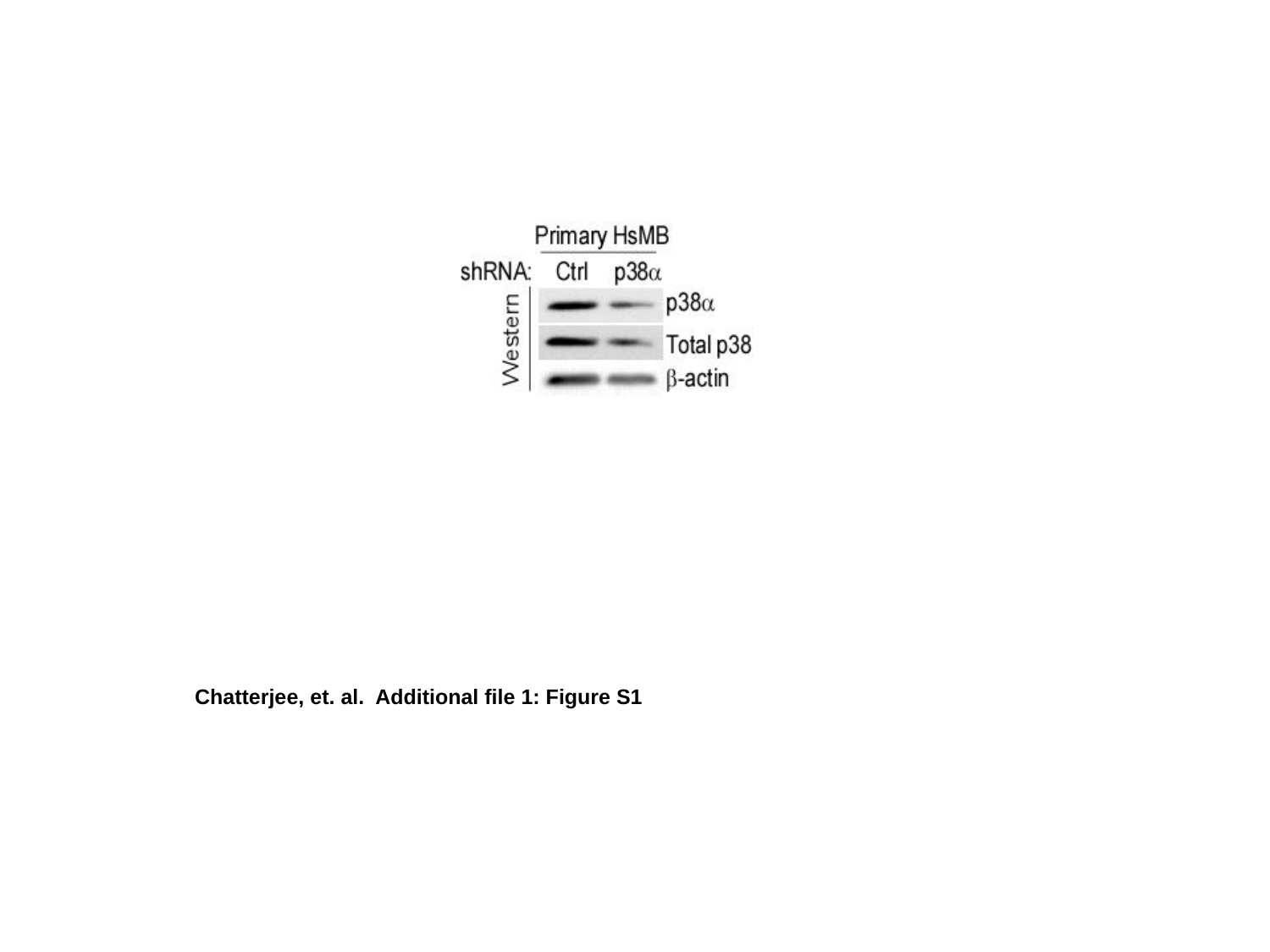

Chatterjee, et. al. Additional file 1: Figure S1

Supplement: Additional file 1: Figure S1. — Knockdown of p38α was monitored by western blot analysis of cell extracts from primary HsMB cells expressing control scramble shRNA (Ctrl) or p38α shRNA, probed with antibodies for p38α, total p38, β-actin as loading control. Decreased levels of both p38α and total p38 were observed by p38α shRNA relative to Ctrl shRNA. (PPTX 47 kb) [file 13395_2016_100_MOESM1_ESM.pptx]

## Slide 1
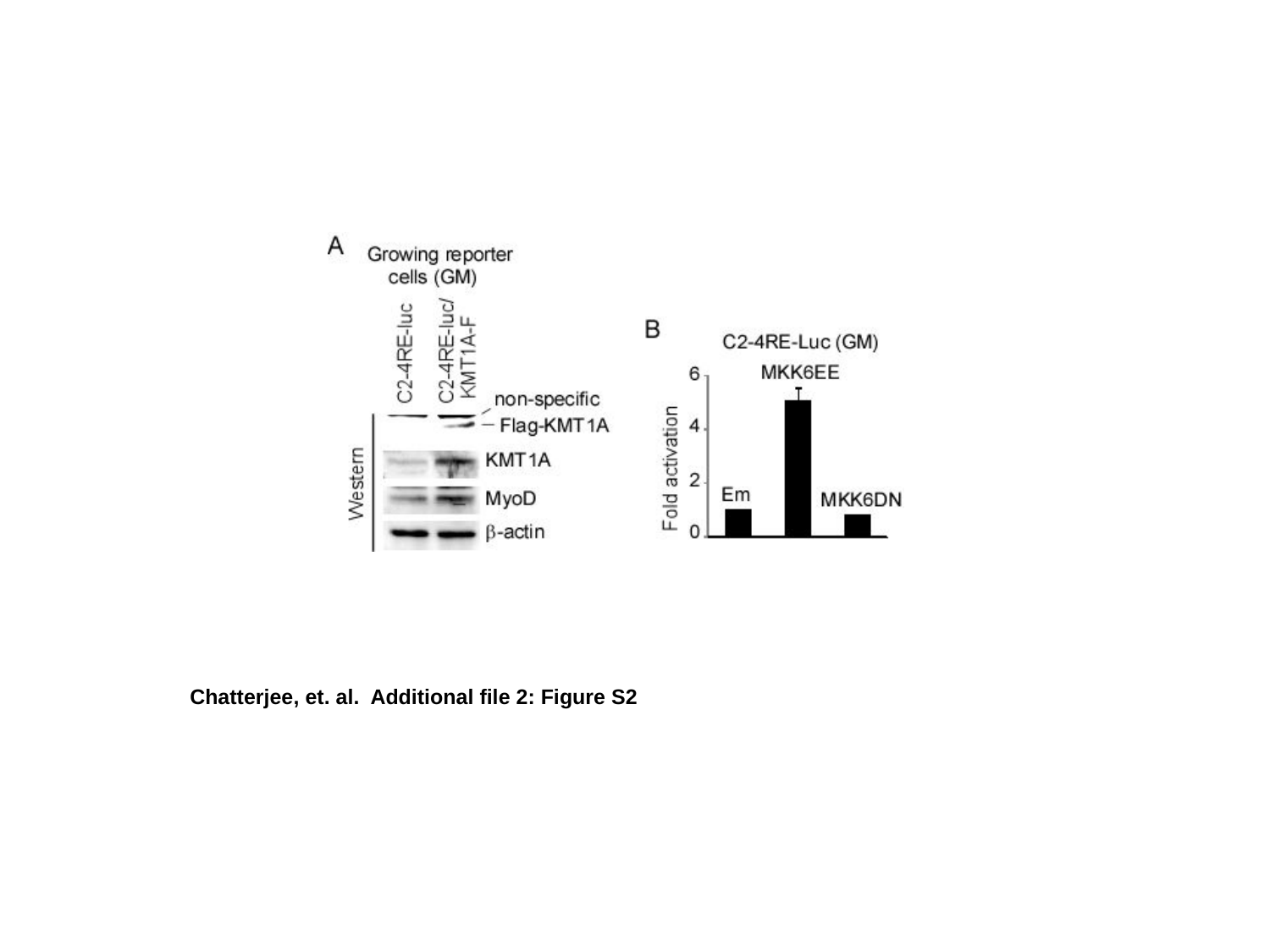

Chatterjee, et. al. Additional file 2: Figure S2

Supplement: Additional file 2: Figure S2. — (A) Ectopic Flag-KMT1A expression was determined by western blot analysis of C2-4RE-luc and C2-4RE-Luc/KMT1A-F cells grown in GM, probed with antibodies to Flag-KMT1A, total KMT1A, MyoD, and β-actin as loading control. Flag-KMT1A expression was observed only in C2-4RE-luc/KMT1A-F cells. (B) Luciferase activity was monitored in C2-4RE-luc cells expressing vector control (Em), MKK6EE or MMK6DN grown in GM and values expressed after protein normalization as fold activation. Error bar, ±SEM (n = 3). MyoD-responsive reporter luciferase gene activation was observed by MKK6EE but not MKKDN in these cells. (PPTX 59 kb) [file 13395_2016_100_MOESM2_ESM.pptx]

## Slide 1
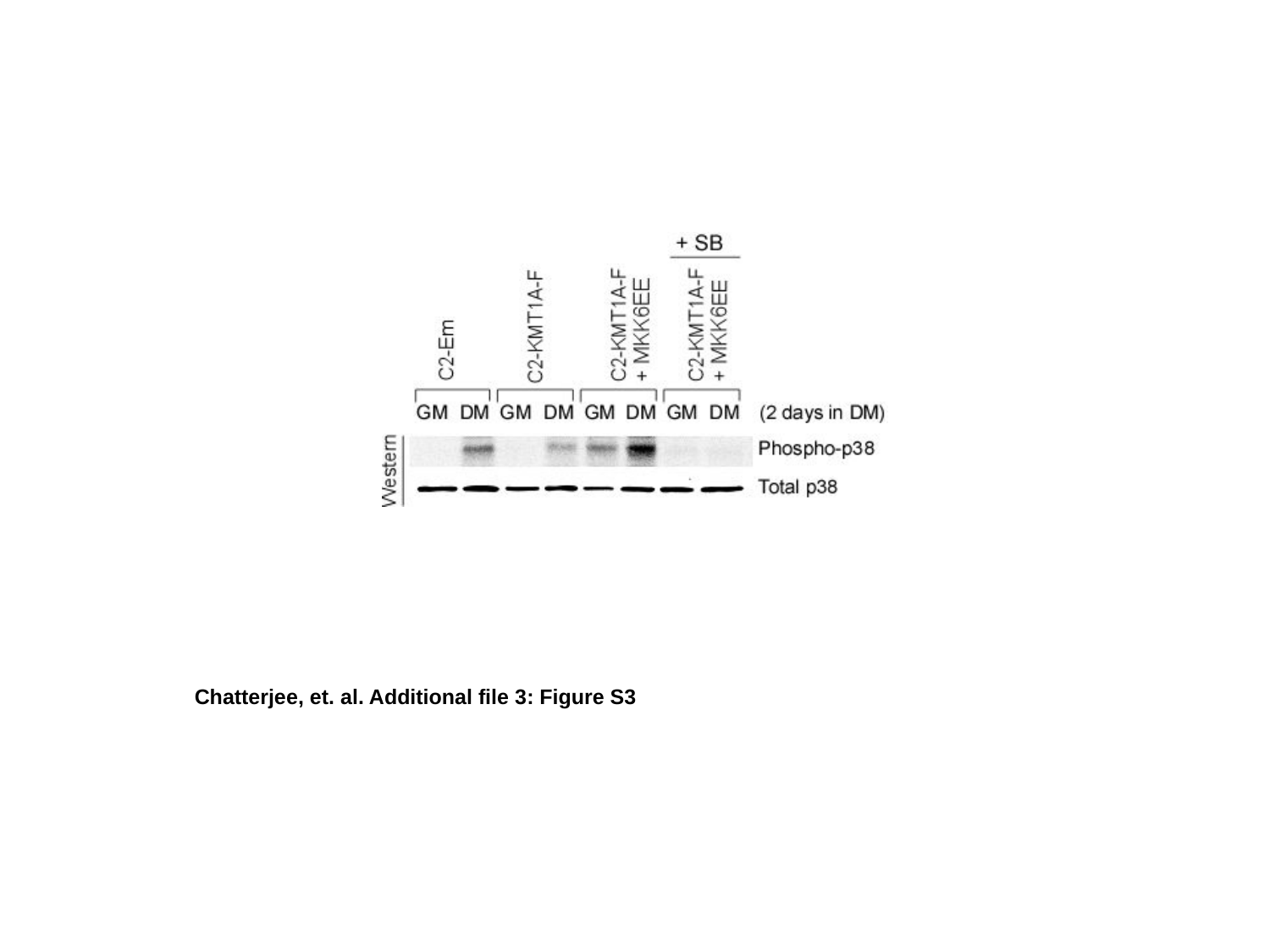

Chatterjee, et. al. Additional file 3: Figure S3

Supplement: Additional file 3: Figure S3. — The levels of phosphor-p38 as an indicator of its activated status was monitored by western blot analysis of indicated cells grown in GM or DM in the presence or absence of SB, probed with anti-phospho-p38 and anti-p38 antibodies, where the later antibodies used for monitoring the equivalent levels of total p38 in cell extracts. (PPTX 57 kb) [file 13395_2016_100_MOESM3_ESM.pptx]

## Slide 1
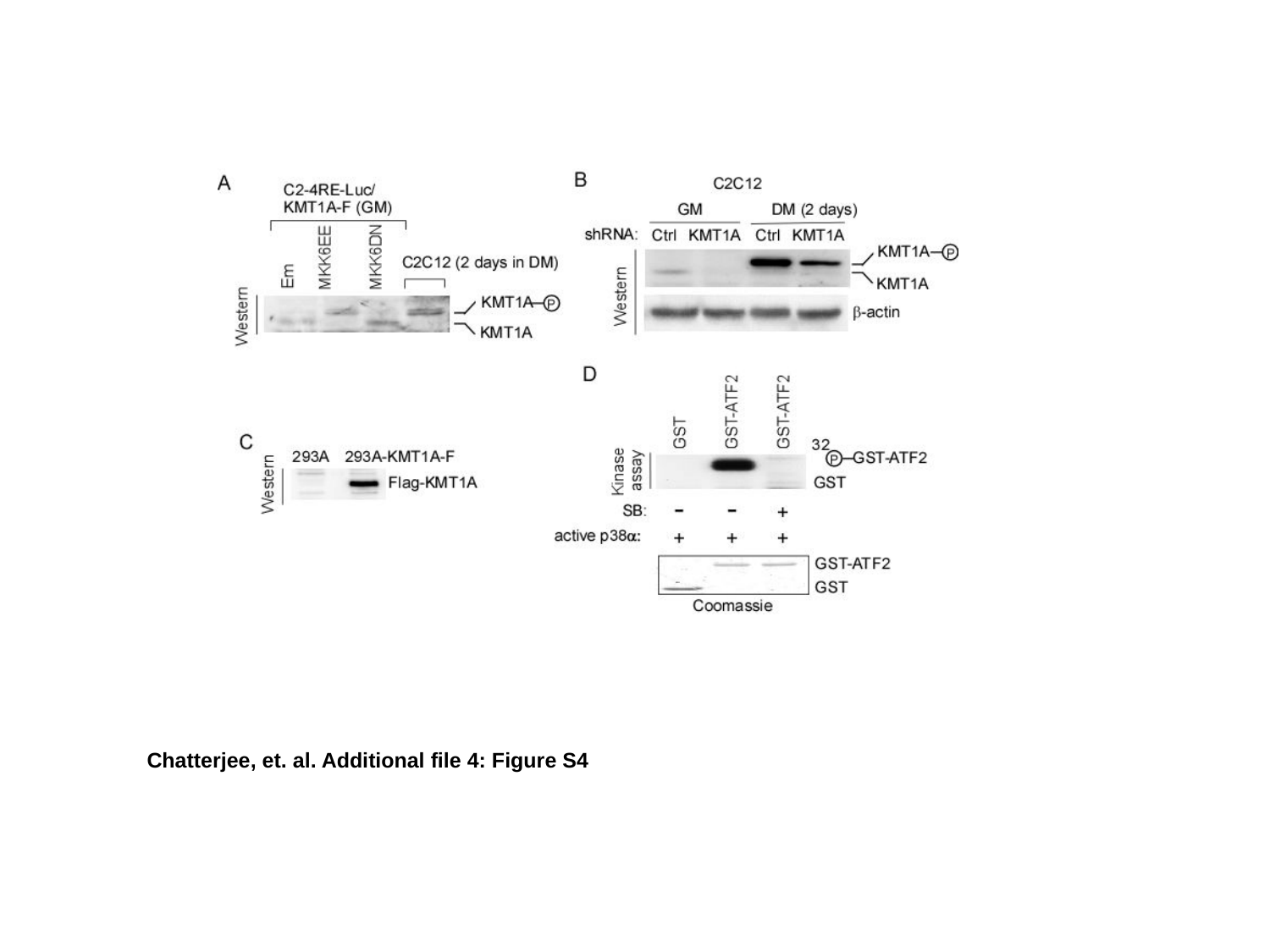

Chatterjee, et. al. Additional file 4: Figure S4

Supplement: Additional file 4: Figure S4. — Western blot analysis verified phosphorylated KMT1A by re-probing the membrane of the western blot results presented in Fig. 4a with a separate KMT1A antibody. (B) Western blot analysis of C2C12 cells expressing control scramble shRNA (Ctrl) or KMT1A shRNA via lentiviral delivery grown in GM or DM, probed with antibodies against KMT1A, and β-actin as loading control. Decreased levels of both under- and phosphorylated KMT1A were observed by KMT1A shRNA relative to Ctrl. (C) Flag-KMT1A overexpression was monitored by western blot analysis of cell extracts following it expression in 293A cells (293A-KMT1A-F) via lentiviral delivery. (D) In vitro kinase assays was performed for p38α activity using GST or GST-ATF2 as substrate by in vitro kinase assays in the presence and absence of SB. Commassie and autoradiography detected inputs GST/GST-ATF2 proteins and phosphorylated ATF2, respectively. (PPTX 99 kb) [file 13395_2016_100_MOESM4_ESM.pptx]

## Slide 1
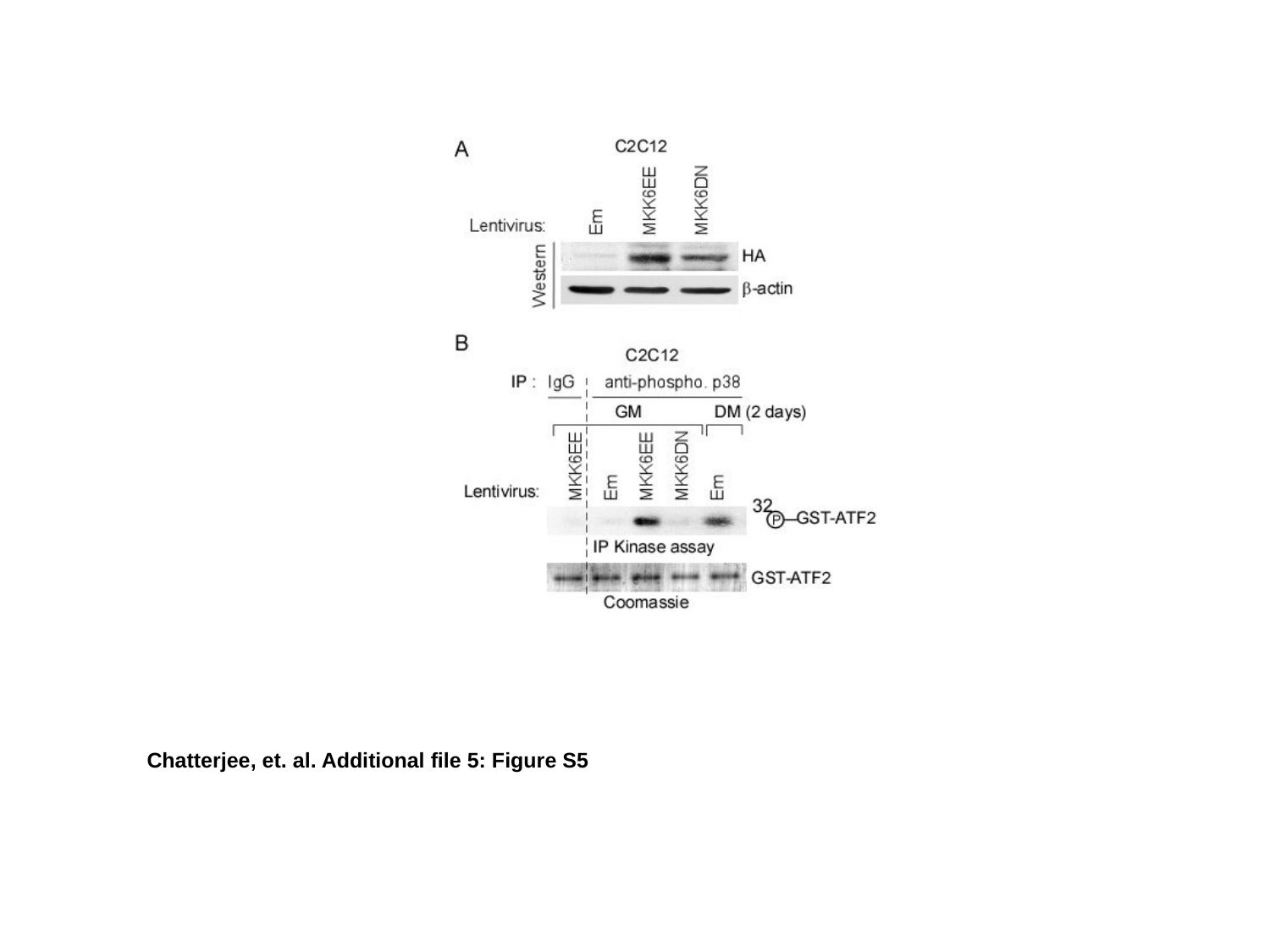

Chatterjee, et. al. Additional file 5: Figure S5

Supplement: Additional file 5: Figure S5. — Expression of HA-tagged MKK6EE and MKK6DN in C2C12 cells was verified by western blot analysis, probed with antibodies against HA, and β-actin as loading control. (B) Control IgG or anti-phospho-p38 immunoprecipitates retrieved from extracts of indicated cells grown in GM or DM were subjected to in vitro kinase assays to monitor p38 activation using GST-ATF2 as substrate. Autoradiography and Commassie detected phosphorylated ATF2 and GST-ATF2 protein, respectively. (PPTX 75 kb) [file 13395_2016_100_MOESM5_ESM.pptx]
